# 

**Published:** 1894-12-01

**Authors:** 


					THE hospital. ? /1 Dec- l?1894-
PRICE TWOPENCE.
WITH EXTRA NURSING SUPPLEMENT.
* No. 427, Vol"XVN.?Dec."Vt,~1894. g% JT
t^&istered at the General Post Office as a Newspaper.] Ditto per Annum, 8s. 6d. post free,
x>
No. 42^ Vol. XVII. WITH EXTRA NURSING SUPPLEMENT. Saturday,
Dec 1st, 1891.

				

